# Influence of time under mechanical ventilation on bronchopulmonary dysplasia severity in extremely preterm infants: a pilot study

**DOI:** 10.1186/s12887-020-02129-2

**Published:** 2020-05-21

**Authors:** Victoria Escobar, Darllyana S. Soares, Jane Kreling, Ligia S. L. Ferrari, Josiane M. Felcar, Carlos A. M. Camillo, Vanessa S. Probst

**Affiliations:** 1 Neonatal Intensive Unit, University Hospital, Londrina State University (UEL), Londrina, PR Brazil; 2 Postgraduation Program in Rehabilitation Sciences UEL-UNOPAR, Londrina State University (UEL), Londrina, PR Brazil; 3 Pediatric Surgery and Pediatric Department, Center of Health Sciences, Londrina State University (UEL), Londrina, PR Brazil; 4 Physiotherapy Department, Center of Health Sciences, Londrina State University, 60 Robert Koch Av, Londrina, Parana 86038-350 Brazil; 5 Pitagoras Unopar University, Londrina, PR Brazil

**Keywords:** Premature, Bronchopulmonary dysplasia, Mechanical ventilation

## Abstract

**Background:**

The relation between mechanical ventilation (MV) and bronchopulmonary dysplasia (BPD) - a common disease in extremely premature newborn (PTNB) - is well stabilished, but is unknown, however, how much time under MV influences the severity of the disease.

**Aim:**

To define the duration under MV with greater chance to develop moderate to severe BPD in extremely PTNB and to compare clinical outcomes before and during hospitalization among patients with mild and moderate to severe BPD.

**Methods:**

Fifty-three PTNB were separated into mild and moderate to severe BPD groups and their data were analyzed. Time under MV with a greater chance of developing moderate to severe BPD was estimated by the ROC curve. Perinatal and hospitalization outcomes were compared between groups. A logistic regression was performed to verify the influence of variables associated to moderate to severe BPD development, such as pulmonary hypertension (PH), gender, gestational age (GA) and weight at birth, as well the time under MV found with ROC curve. The result of ROC curve was validated using an independent sample (*n* = 16) by Chi-square test.

**Results:**

Time under MV related to a greater chance of developing moderate to severe BPD was 36 days. Moderate to severe BPD group had more males (14 vs 5, *p* = 0,047), longer time under MV (43 vs 19 days, *p* < 0,001), more individuals with PH (12 vs 3, *p* = 0,016), worse retinopathy of prematurity (grade 3, 2 vs 11, *p* = 0,003), longer hospital length of stay (109 vs 81,5 days, *p* < 0,001), greater PMA (41 vs 38 weeks, *p* < 0,001) and weight (2620 vs 2031 g, *p* < 0,001) at discharge and the mild BPD group had more CPAP use prior to MV (12 vs 7, *p* = 0,043). Among all variables included in logistic regression, only PH and MV < 36 days were significant in the model, explaining 72% of variation in moderate to severe BPD development. In the validation sample, prevalence of preterm infants who needed MV for more than 36 days in the moderate to severe BPD group was 100% (*n* = 6) and 0% in mild BPD group (*p* = 0,0001).

**Conclusion:**

Time under MV related to moderate to severe BPD development is 36 days, and worst outcomes are related to disease severity. PH and time under MV for more than 36 days are related to development of moderate to severe BPD.

## Background

Bronchopulmonary dysplasia (BPD) is a multifactorial disease that occurs due to interactions among exposures during pregnancy, injuries from oxygen use and postnatal invasive mechanical ventilation (MV), as well as other injuries such as infections and inadequate nutrition after birth [1]. Diagnosis and classification remain uncertain, as there is still no consensus on the ideal postmenstrual age (PMA) for assessment of oxygen-dependent premature newborns. The 2000 National Heart, Lung and Blood Institute workshop recommends that for preterm infants with gestational age less than 32 weeks, BPD is defined as oxygen exposure for ≥28 days. It can also be categorized as mild (in room air at 36 weeks of PMA or at discharge - whichever comes first), moderate (supplemental oxygen at 36 weeks of PMA or at discharge - whichever comes first), or severe (supplemental oxygen ≥30% and/or positive pressure at 36 weeks of PMA) [2]. BPD is a morbidity associated with extreme prematurity, and it has now been debated that milder forms of BPD do not seem to have severe consequences, such as serious respiratory morbidity and neurosensory impairment [1, 3].

Extremely preterm infants, those born at < 28 weeks of PMA [4], are frequently submitted to MV [5], which despite being a necessary and widely used treatment, has well established adverse effects. The risk of death is 8 times higher in extremely low birth weight preterm infants undergoing MV for more than 6 weeks compared to those exposed only to 7 days or less of invasive ventilation. This risk increases to 13 times when isolated only to cardiorespiratory causes [6]. Morbidities such as pneumothorax, ventilator-associated pneumonia, retinopathy of prematurity (ROP) requiring surgical intervention and even neurodevelopmental impairments are also associated with MV in preterm infants [7–9]. In addition, duration of MV is a strong predictor for BPD development: each additional week increases 2.7 times the odds for BPD [10]. Although the relationship between invasive mechanical ventilation and the development of the disease is well established, the duration of MV that interferes with severity classification of BPD remains unknown.

Therefore, the aim of this study was to determine how many days of mechanical ventilation has a greater chance of developing moderate and severe bronchopulmonary dysplasia. Moreover, we aimed to compare clinical variables during hospitalization and prenatal data between patients with mild and moderate to severe dysplasia.

## Methods

### Design and sample

This was a retrospective cohort study (with clinical data prospectively entered in a national database) which was conducted at the University Hospital of the Londrina State University – Brazil. All preterm infants born at ≤28 weeks PMA between January 2015 and December 2017, admitted to the neonatal unit who survived for more than 28 days and were diagnosed with BPD were eligible. Infants who did not use invasive mechanical ventilation during hospitalization and those who died before 36 weeks of PMA were excluded. This was considered as a pilot study since we assessed data from only one hospital which participates in the Brazilian Neonatal Research Network. The study was approved by the Research Ethics Commitee Involving Human Beings – Londrina State University – Brazil (commitee’s reference number: 3.362.155).

### Procedures

Patients included in the study were allocated into two groups: (I) mild BPD, those with diagnosis of BPD breathing in room air at 36 weeks of PMA; and (II) moderate to severe BPD, preterm infants diagnosed with BPD who required oxygen or some type of ventilatory support at 36 weeks of PMA. The definition of BPD and its severity were described by Jobe and Bancalari [2].

#### Perinatal variables were didactically divided into four groups

- *Maternal and gestational characteristics*: use of tobacco during pregnancy, use of antenatal corticosteroids and clinical diagnosis of chorioamnionitis;
- *Perinatal variables*: postmenstrual age - weeks and days of gestation determined by ultrasound in the first trimester or date of last menstruation - birth weight, gender, Apgar score at 5th minute of life and type of stabilization required in the delivery room - intubation or noninvasive ventilation through continuous positive airway pressure – CPAP;
- *Outcomes during hospitalization*: time under invasive mechanical ventilation, CPAP use before intubation, surfactant admnistration, vasoactive drugs (VADs) until the third day of life, degree of retinopathy of prematurity [11] and need for surgical correction - diagnosed by an ophthalmologist by retinal examination, patent ductus arteriosus (PDA) - diagnosed by a pediatric cardiologist by echocardiography - and type of treatment - drug or surgical, also indicated by cardiologist, pulmonary hypertension (PH) - also diagnosed by a pediatric cardiologist on echocardiography - clinical diagnosis of necrotizing enterocolitis (NEC) [12], pneumonia, pneumothorax and sepsis [13];
- *Variables at discharge*: length of stay, weight and PMA at discharge.

#### Data on invasive mechanical ventilation

The two main ventilation modes used in the study were time-cycled pressured-limited continuous flow ventilation and target volume ventilation. High-frequency oscillatory ventilation was rarely used. Extubation criteria included arterial blood gases within normal limits and ventilator parameters that were considered low, in addition to the stability of the newborn.

### Statistical analysis

Statistical analysis was performed using SPSS Statistics 22 (IBM Corp, Armonk, NY) and Graph Pad Prism 6.0 software (GraphPad Software Inc.; San Diego, California, USA). Data distribution analysis was performed by Shapiro-Wilk test and data were described as percentages and mean ± standard deviation or median and interquartile range, according to the normality test. To estimate time under mechanical ventilation to predispose the highest chance of developing moderate to severe BPD, a ROC curve with time under mechanical ventilation was generated in days for both groups, and the best combination of sensitivity and specificity was used (index of Youden [14]). The area under the ROC curve (AUC) is a measure of cutoff point accuracy. AUC ROC values ≥0.90 are considered excellent, 0.80–0.89 good, 0.70–0.79 reasonable and < 0.70 are considered poor [15]. Comparison of variables between groups was performed by Student’s t-test or Mann-Whitney’s test for continuous data, and proportion measurements were analyzed using the Chi-square test. For comparing ROP degrees between groups, post hoc analysis involved pairwise comparisons using multiple z-tests of two-proportion with a Bonferroni correction, and statistical significance was accepted at *p* < 0.006. A logistic regression analysis was performed to verify the influence of variables previously known by their relationship with BPD development (such as PMA at birth, birth weight, gender and diagnosis of PH), as well as time under MV found by the ROC curve in the development of moderate to severe BPD. Variables that showed standard deviation greater than 2.5 in residual analysis were excluded. In order to validate the cut-off point found by ROC curve, Chi-square test was performed on a different sample (*n* = 16) from the previously analyzed, applying the same eligibility, inclusion and exclusion criteria of the primary analysis with patients born in 2018 and 2019, using the time under MV above and below the cutoff point in each group. The statistical significance adopted was *p* < 0.05.

## Results

**Fig. 1:**
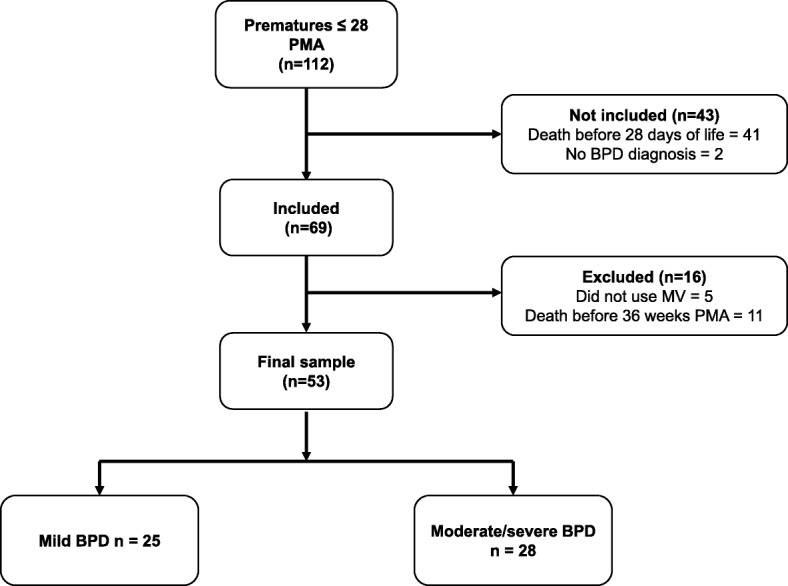
Flowchart of the study

Of the 112 preterm infants born at ≤28 weeks PMA admitted to the unit, 41 died before completing 28 days of life and 2 were not diagnosed with BPD. Among those included in the study, 16 were excluded: 5 did not need MV during hospitalization and 11 died before 36 weeks PMA, making BPD classification not possible (Fig. 1). Data such as PMA at birth, weight, gender, Apgar score, MV time and length of stay of patients included in the study are described in Table 1.

**Table 1:** Characteristics of patients included in the study

Characteristics	Mild BPD group (***n*** = 25)	Moderate/severe BPD group (***n*** = 28)	***p***
GA, weeks	26,34 ± 1,02	25,92 ± 1,37	0,305
Weight at birth, g	830,5 ± 150,26	760,07 ± 166,04	0,119
Gender, M (%)	5 (20)	14 (50)	0,047*
Apgar 5′	8 [6–9]	8 [7–8]	0,81

*GA* gestational age, *g* grams, *M* male, *Apgar 5′* Apgar score at fifth minute of life; * *p* < 0,05.

**Fig. 2:**
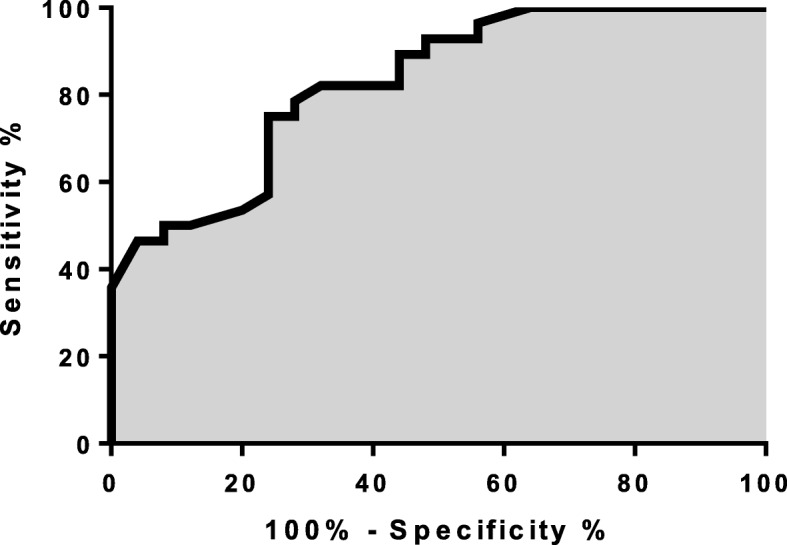
Cut-off point of time under mechanical ventilation. ROC curve showing sensibility and specificity of time under MV with greater chance to develop moderate/severe BPD. Area under the curve 0,83

The cutoff point for time under MV identified as the best predictor for moderate to severe BPD development identified on the ROC curve was 36 days (75% sensitivity [95% CI 55 to 89] and 76% specificity [95% CI 55 to 91]). Area under the curve was 0.83 (Fig. 2).

The comparison between the groups of preterm infants with mild BPD and those with moderate to severe BPD can be found in Table 2. There was no difference between groups regarding maternal and gestational characteristics. Both groups were similar in weight, PMA at birth, Apgar score and respiratory support needed in the delivery room, but the moderate to severe BPD group had a higher proportion of males.

**Table 2:** Comparison between groups

	Mild BPD	Moderate/severe BPD	***p***
**Maternal and gestational characteristics**			
Tobacco use, yes (%)	2 (8)	4 (14,3)	0,672
Chorioamnionitis, yes (%)	3 (12)	5 (18,5)	0,705
Antenatal corticosteroids, yes (%)	23 (92)	20 (74,1)	0,143
**Perinatal variables**			
Intubation in the delivery room, yes (%)	12 (48)	20 (71,4)	0,072
CPAP in the delivery room, yes (%)	13 (52)	8 (28,6)	0,99
**Outcomes during hospitalization**			
Time under MV (days)	19 [7,25–38]	43 [33,25-52,75]	< 0,001*
CPAP before intubation, yes (%)	12 (56)	7 (25)	0,043*
RDS, yes (%)	13 (76)	24 (85,7)	0,488
Surfactant use, yes (%)	18 (72)	24 (85,7)	0,313
Pulmonary hypertension, yes (%)	3 (12)	12 (42,9)	0,016*
Pneumonia, yes (%)	23 (92)	28 (100)	0,218
ROP classification, n (%)			
0	13 (52)	4 (14,3)	0,009
1	6 (16)	11 (39,3)	0,23
2	4 (16,2)	2 (7,1)	0,31
3	2 (8)	11 (39,3)	0,003^a^
Corrective surgery for ROP, yes (%)	1 (5,6)	4 (15,4)	0,634
PDA, sim (%)	22 (88)	24 (85,7)	1
Drug treatment for PDA, yes (%)	9 (40,9)	11 (45,8)	0,774
Surgery for PCA, yes (%)	2 (6,5)	1 (4,2)	0,6
NEC, yes (%)	3 (12)	3 (10,7)	1
Surgery for NEC, yes (%)	1 (33,3)	2 (66,7)	1
Sepsis, yes (%)	22 (88)	26 (92,9)	0,658
VAD, yes (%)	5 (20)	2 (7,1)	0,234
**Variables at discharge**			
Lenght of stay (days)	81,5 [69,5-90,5]	109 [99–118,25]	< 0,001*
GA at discharge (weeks)	38 [36,25-38,75]	41 [39–42,75]	< 0,001*
Weight at discharge (g)	2031 [1868–2328]	2620 [2045–3030]	< 0,001*
O_2_ discharge/transference, yes (%)	0 (0)	4 (14,3)	0,115

*GA* gestational age, *g* grams, *Apgar 5*′ Apgar score at fifth minute of life, *M* male, *MV* mechanical ventilation, *CPAP* continuous positive airway pressure, *RDS* respiratory distress syndrome, *ROP* retinopathy of prematurity, *PDA* patente ductus arteriosus, *NEC* necrotizing enterocolits, *VAD* vasoactive drugs, *O*_*2*_ oxygen; * *p* < 0,05; ^a^*p* < 0,006

Regarding the variables during hospitalization, preterm infants in moderate to severe BPD group had a longer time under MV, lower rates of CPAP use before intubation, higher rates of pulmonary hypertension, and greater severity of ROP. At discharge, individuals in the moderate to severe BPD group had been hospitalized longer and had a higher weight and were older than those in the mild BPD group.

Logistic regression which verified the influence of different clinical variables in the development of moderate to severe BPD was statistically significant (*p* < 0.0005). The model explained 72% of the variance in moderate to severe BPD and correctly classified 86% of the cases. After checking for residuals, three subjects were excluded from the analysis. Only two out of the five predictor variables were statistically relevant: PH and time under MV greater than 36 days (Table 3). Preterm infants who remained under MV for more than 36 days were 49 times more likely to develop moderate to severe BPD than those who needed MV for less than 36 days. The diagnosis of PH was associated with 37 times increase in chances of developing moderate and severe BPD.

**Table 3:** Logistic Regression predicting likelihood of developing moderate/severe BPD based on MV > 36 days, GA, PH, weight and gender

				95% C.I. for EXP	
Variable	B	***p***	RR	Lower	Upper
Constant	− 7528	0,506	0,001		
MV > 36 days	3894	0,001	49,101	4916	490,415
GA (days)	0,048	0,469	1050	0,921	1196
PH	3632	0,011	37,770	2270	628,541
Weight at birth	−0,005	0,125	0,995	0,988	1001
Gender	2014	0,143	7497	0,506	110,976

*MV* Mechanical ventilation, *GA* gestational age, *PH* Pulmonary hypertension, *B* nonstandard coefficients regression, *RR* relative risk, *C.I* confidence interval. *Note*: Gender is for males compared to females

In the validation sample, of the 16 preterm included in the analysis, 10 had mild BPD and 6 moderate to severe BPD. The prevalence of preterm infants who were under MV for more than 36 days in the moderate to severe BPD group was 100% (*n* = 6), and 0% in the mild BPD group (*p* = 0.0001).

## Discussion

The present study showed that there is greater chance of developing moderate to severe BPD in extremely premature newborns after 36 days under mechanical ventilation [16]. It is important though to make it clear that BPD is a multifactorial disease, and its development is also associated to factors such as intrauterine infections, growth restriction and nicotine exposure [16]. Due to the pathophysiology of the disease, BPD remains the most frequent morbidity in extremely premature infant [17], and the diagnosis of moderate to severe BPD is associated with worse prognosis. A previous study comparing infants with different disease severity classifications showed that the group with moderate to severe BPD had a higher proportion of subjects with grade 3 and 4 intraventricular hemorrhage, periventricular leukomalacia, NEC, late sepsis, home oxygen use and death after discharge, in addition to the increased incidence of neurological impairment, worse mental and psychomotor development, blindness and hearing impairment [18]. In the present study, infants with moderate to severe BPD had worse ROP, higher prevalence of PH, and longer length of hospital stay, corroborating the findings in the literature that preterm infants have worse outcomes.

Studies have shown a high incidence of moderate to severe BPD in extremely premature infants (PMA up to 27-29w). Stoll et al. reported an incidence of 41% in their population [19]. Another study with extremely low birth weight infants (401–1000 g) found that 52% of the population had diagnosis of moderate to severe BPD [18]. In the present study, more than half of the preterm infants included (53%) had moderate to severe BPD (Fig. 1).

Recent studies have enhanced the harms of exposure for extremely preterm infants to MV for long periods of time. Yossef et al. observed that a group of extremely premature infants that were submitted to more than 56 days of MV had a higher incidence of moderate to severe BPD [20]. Choi et al. found an almost three-fold increase in the risk of mortality of extremely low birth weight preterm infants who required MV for 15 to 28 days. Furthermore, that study also associated cumulative duration of MV with ROP requiring surgical correction, neurological impairment, BPD, PH, and length of stay [6]. The major cause of the development of ROP is the exposure of preterm infants to supplemental oxygen therapy at high concentrations, therefore its relationship with prolonged MV is already expected [11]. Although the number of days under MV found in our study is different from the aforementioned studies, our findings are mostly similar to the literature. It reinforces the importance to avoid invasive MV, as well as to limit the duration that the extremely preterm infants are exposed to this treatment.

The mild BPD group had more individuals who used CPAP before MV, and it is an expected result. In centers where CPAP is used during stabilization in the delivery room, the incidence of severe BPD is 3.3% [21], and intubation for surfactant administration alone (without MV) has shown a significant reduction in the use of MV in extremely low birth weight preterm infants [22]. In addition, compared with intubation right after birth, CPAP reduces the incidence of BPD and death at 36 weeks of PMA [23].

Laughon et al. in their large study (*n* = 3629 preterm infants) to develop an instrument for predicting BPD risk and death through clinical information, six variables were relevant to the model: PMA at birth, birth weight, ethnicity, gender, respiratory support and inspiratory oxygen fraction on specific days of hospitalization [10]. Factors such as PMA and weight were also related to longer time under MV^20^. In our search for predictors for development of moderate to severe BPD, in addition to time on MV greater than 36 days, the presence of PH appeared as significant in the regression model. It is associated with prolonged exposure to MV^6^, and the association between PH and BPD is related to both its pathogenesis (similarly associated to complications of prematurity) and secondary PH development in BPD [24]. There was no difference between groups regarding PDA, NEC, use of VAD, chorioamnionitis and maternal use of tobacco, unlike other studies about both prolonged time under MV and higher severity of BPD [6, 20], which might have occurred due to the relatively small sample size. Additionally, a reasonable number of BPD patients did not survive until they completed 36 weeks PMA, which may also have contributed to this difference in outcomes.

Controversies regarding the definition and classification of BPD in literature may have limited our results. One premature newborn died before completing 36 weeks PMA and 41 died before 28 days of age. According to the most recent workshop on BPD, those infants could be classified as BPD type IIIa (early death from lung disease and respiratory failure) [25], and this could have influenced our findings. Our study involved preterm infants from a single institution and included a small sample compared to similar ones. Additionally, long-term outcomes such as neurodevelopment were not analyzed. However, almost all variables that may influence the development and severity of BPD were analyzed, except genetic factors. Despite the weaknesses, most of the findings of the present study, which is a pilot study, are in line with the findings from the literature.

Despite the limitations, it is noteworthy that this study is the first to investigate a cutoff point of time under MV to identify the chance of developing more severe forms of BPD. In addition, the result found was validated in an independent sample. Our study was able to define 36 days as a time of MV exposure that is associated with the development of moderate to severe BPD and, as a consequence, may have worse outcomes, such as greater severity of ROP and length of hospital stay. Therefore, it is suggested that MV should be avoided whenever possible, using strategies such as prophylactic CPAP and less invasive surfactant admnistration [26, 27]. Moreover, MV should be interrupted as soon as possible, preferably before 36 days to reduce the risk of developing moderate to severe BPD, as well as to avoid impairments associated with the severity of the disease. Furthermore, a multicenter study should be performed to verify whether similar results are observed with a larger sample of patients. Indeed, this characterizes the next step for a future study in which we will include other hospitals from the Brazilian neonatal research network.

## Conclusion

In conclusion, we can state that the duration of MV exposure associated with the development of moderate to severe BPD was 36 days, and worse outcomes such as worse ROP and longer hospital staying are associated with disease severity.

## Data Availability

The datasets used and/or analysed during the current study are available from the corresponding author on reasonable request.

## Abbreviation

BPD: Bronchopulmonary dysplasia
MV: Mechanical Ventilation
GA: Gestational age
ROP: Retinopathy of prematurity
CPAP: Continuous positive airway pressure
VAD: Vasoactive drugs
PDA: Patent ductus arteriosus
PH: Pulmonary Hypertension
NEC: Necrotizing enterocolitis
AUC: Area under the curve
